# Validity of a Short Food Frequency Questionnaire for Toddlers of NELA Birth Cohort Study

**DOI:** 10.3390/nu16244403

**Published:** 2024-12-22

**Authors:** Sandra Gonzalez-Palacios, Carolina Ojeda-Belokon, Alejandro Oncina-Canovas, Laura-María Compañ-Gabucio, María-Teresa Pastor-Fajardo, Adrian Pérez, Antonio Gázquez, Elvira Larqué, Eva Morales, Jesús Vioque, on behalf of the NELA Study Group

**Affiliations:** 1 Unidad de Epidemiología de la Nutrición (EPINUT), Departamento de SaludPública, Historia de la Ciencia y Ginecología, Universidad Miguel Hernández (UMH), 03550 Alicante, Spain; sandra.gonzalezp@umh.es (S.G.-P.); cojeda@umh.es (C.O.-B.); aoncina@umh.es (A.O.-C.); lcompan@umh.es (L.-M.C.-G.); 2 Instituto de Investigación Sanitaria y Biomédica de Alicante (ISABIAL), 03010 Alicante, Spain; 3 Consortium for Biomedical Research in Epidemiology and Public Health (CIBERESP), 28029 Madrid, Spain; evamorales@um.es; 4 Department of Pediatrics, Hospital General Universitario de Elche, Elche, 03203 Alicante, Spain; pastormariate@gmail.com; 5 Biomedical Research Institute of Murcia (IMIB-Arrixaca), 30120 Murcia, Spain; antonio.gazquez@um.es (A.G.); elvirada@um.es (E.L.); 6 Departament of Fisiology, University of Murcia, 30100 Murcia, Spain; adrian.perez@um.es; 7 Division of Preventive Medicine and Public Health, Department of Public Health Sciences, Faculty of Medicine, University of Murcia, 30003 Murcia, Spain

**Keywords:** validity, reproducibility, food frequency questionnaire, toddler, nutrients, food groups, intakes

## Abstract

Background/Objectives: Our aim was to evaluate the reproducibility and validity of a semi-quantitative food frequency questionnaire (FFQ) for the assessment of usual nutrient and food intakes in children of 18 months old. Methods: We included 103 toddlers aged 18 months from the Nutrition in Early Life and Asthma (NELA) birth cohort study. A 47-item FFQ was administered twice to parents with a 3-month interval. During that period, we also performed three non-consecutive 24 h dietary recalls (24hDRs) and oral mucosa samples for determining the fatty acid profile (glycerophospholipids). We estimated correlation coefficients of reproducibility for nutrient and food group intakes by comparing both FFQs and validity coefficients by comparing nutrient intakes between the second FFQ and the average of the three 24hDRs. We also explored biochemical validity by comparing the intake of fish from the FFQ with the percentage of fatty acids in oral mucosa cells. Results: The average of correlation coefficients for FFQ reproducibility was r = 0.48 for energy-adjusted nutrient intakes (ranging from r = 0.28 for Na to r = 0.62 for Mg and Zn) and r = 0.35 for the intake of energy-adjusted food groups. The average correlation coefficient for FFQ validity on nutrient intakes was r = 0.48, ranging from r = 0.16 for α-carotene to r = 0.75 for vitamin E. We also found a positive correlation between total omega-3 fatty acids and docosahexaenoic acid percentages in oral mucosa cells and the intake of total and white fish, r = 0.31 and r = 0.34, respectively. Conclusions: This study shows that our short FFQ demonstrated moderate reproducibility (mean r = 0.48) and validity (mean r = 0.48) for dietary assessment of most nutrients and foods in 18-month-old children in Spain. This FFQ provides an efficient and minimally invasive method for evaluating toddler dietary intake, particularly in Mediterranean contexts.

## 1. Introduction

Dietary habits in early childhood significantly influence long-term health, necessitating valid and age-appropriate assessment tools [1,2]. Nevertheless, there is no perfect dietary assessment method to measure diet in young children [3]. The selection of a dietary assessment method depends on multiple factors, including the study objective, the available time, or budget. In this sense, food frequency questionnaires (FFQs) have been considered the most appropriate dietary assessment method and, consequently, are widely used in epidemiological studies because they are fast and economical and allow the evaluation of the usual intake of foods and nutrients in large population samples [4]. However, reproducibility and validity of FFQs must be evaluated in each study population to ensure a reliable dietary assessment [5].

Validation of FFQs in younger children presents challenges. During the first years of life, children experience an evolution in their eating patterns, characterized by a change from a milk-based diet to one that includes a wider variety of foods and textures [6,7]. Few validated FFQs exist for toddlers [8,9,10,11,12,13,14,15,16,17], likely due to challenges in assessing rapidly evolving dietary patterns. Previous studies report correlation coefficients ranging from 0.30 to 0.53, when their nutrient intakes were compared with those from other methods of reference such as dietary records or 24 h dietary recalls (24hDRs) [8,9,10,11,12,13,14,15,16,17]. However, we are not aware of any FFQs validated in toddlers from Mediterranean countries.

In contrast, the number of validated FFQs for older children and adolescents [18] is greater. Most of these validations used the 24hDR as the reference method [18], probably because it is a quick and easy-to-use dietary assessment that provides satisfactory nutrient estimates when administered by expert interviewers following the multiple-pass 24 h recall method [19]. Other studies have used a relative biochemical validation using plasma nutrient biomarkers as a reference method to validate FFQs [10,20,21,22]. However, blood samples are invasive and are less accepted, particularly in infant and toddler populations. Thus, other less invasive methods have been developed such as fatty acid measure from oral mucosa cells, whose precision has been considered comparable to other conventional invasive methods based on plasma fatty acid analyses [23,24].

This study addresses the gap by validating a short FFQ tailored for 18-month-old toddlers, leveraging both dietary recalls and non-invasive biomarkers. Thus, we aimed to evaluate the reproducibility and validity of a short semi-quantitative FFQ to assess usual diet of the previous three months in children aged 18 months who are part of the Nutrition in Early Life and Asthma (NELA) birth cohort study. As reference methods, we used three non-consecutive 24hDRs and the determination of fatty acids in oral mucosa cells.

## 2. Materials and Methods

### 2.1. Study Design and Population

Participants in this study were 103 toddlers from the prospective, population-based, birth cohort NELA study whose parents accepted to participate during the 18-month-old visit. Details of the NELA study have been described previously [25]. Briefly, the main objective of the NELA study was to investigate whether maternal obesity/adiposity and foetal growth and other expositions such as prenatal and postnatal nutrition contribute to the development of asthma in children [25]. Pregnant women were invited to participate in the study in the routine foetal anatomy scan during gestation weeks 19 to 22 at the University Clinical Hospital “Virgen de la Arrixaca” (Murcia, Spain) from March 2015 to April 2018. Since the health care system in Spain is universal and of free access, women and their children in the NELA study share similar characteristics and were representative of women and children from the hospital coverage area [25]. Inclusion criteria were the following: Caucasian and Spanish origin; 18–45 years of age; living in the study area and planning to stay living there for at least 2 years; singleton pregnancy; spontaneous conception; intention to deliver at the University Clinical Hospital ‘Virgen de la Arrixaca; and no major foetal malformations. Exclusion criteria included the following: chronic disease in the mother, such as pregestational diabetes mellitus or other major endocrine disorders, pregestational hypertension, autoimmune disease, or cancer; or verbal communication problems. A total of 1350 pregnant women were invited to participate, and 738 agreed to participate in the programmed follow-up visits of the NELA study. At the visit scheduled at 18 months after childbirth, 532 toddlers were still participating in the follow-up study. Parents were informed about the validation study and sequentially invited to participate. Finally, a total of 103 parents agreed to participate. This sample size was considered satisfactory to detect correlation coefficients as statistically significant [4].

The study was conducted in full compliance with ethical standards, ensuring rigorous participant recruitment and informed consent (Ethical reference Report No. 9/14; 29 September 2014).

### 2.2. Dietary Assessment

A discussion group made up of nutritional epidemiologists (JVL and LMCG) and dietitian–nutritionists (SGP, AOC, and COB) developed the semi-quantitative FFQ for children at the age of 18 months based on a 105-item FFQ previously validated among 4–5-year-old Spanish children, with some modifications [20]. Foods and beverages from the 105-item FFQ (accessible in https://epinut.umh.es/en/cfa-105-inma-infancia/ accessed on 21 December 2024) were grouped into the 47-item FFQ according to their composition and affinity (e.g., the 12 vegetable food items were grouped into two items in the short FFQ). In addition, some food items that were unlikely to be consumed by toddlers were removed (e.g., liver, olives, or nuts), while other food items were added (e.g., formula milk). The final short FFQ consisted of a list of 47 foods, food groups, and beverages with their common portion sizes for toddlers aged 18 months in Spain. Details of food group classifications are available in Supplementary Table S1. The FFQ was administered twice to parents, at baseline and 3 months later. Parents were asked by trained staff (MTPF) about the average food consumption of their offspring during the previous three months. The FFQ had nine possible frequency responses, ranging from “never” to “6 or more times a day”.

The same trained staff (MTPF) that performed the FFQ also collected the information for the three 24hDRs from parents about their offspring’s diet, two related to weekdays and one to a weekend day, using the multiple-pass 24 h recall method [26]. The multiple-pass 24 h recall method is a semi-structured interview technique where participants recall all foods and beverages consumed in the past 24 h in five steps: (1) a quick list of food items consumed, (2) probing for commonly forgotten foods (e.g., snacks, oils), (3) recalling the time of day each item was consumed, (4) providing details on portion sizes, preparation, and ingredients, and (5) a final review to correct any missed or inaccurate items. This method has been shown to provide energy and nutrient estimates similar to more accurate methods, such as weighed food records or doubly labelled water [19,26]. One nutritionist (COB) performed the coding of all food items reported in the three 24hDRs using the Food Processor II software (https://esha.com/products/food-processor/, accessed on 21 December 2024) that allows us to use food composition tables of the US Department of Agriculture [27] and to add specific Spanish foods [28]. We used the average of nutrient intakes from the three 24hDRs as the reference method to explore validity of the FFQ.

### 2.3. Fatty Acids Measured from Oral Mucosa Cell Samples

Oral mucosa cell samples for glycerophospholipid determination (n = 93) were collected from consenting parents during the baseline interview. To ensure that the oral mucosa cell samples were collected under comparable conditions for all children, we follow the protocol described previously in a study with infants [29], although the sampling procedure with toddlers was very challenging to obtain mouth cleaning, drinking 100 mL tap water before sampling, followed by rubbing of each inner cheek side for 20–25 times with an Interprox brush (Ref. 927300626, Dentaid S.L., Barcelona, Spain). Then, the mouth was rinsed with 10 mL pure water, and we collected the rinsing solution containing cells washed off the inner cheeks in a tube. We inserted the brush used for scraping in the tube and stored at −80 °C until analysis [23]. The day of the fatty acid analysis, the tube with the brush was vigorously vortexed, the brush removed, and the tube was centrifuged at 1400× *g* (2710 rpm) for 10 min at 4 °C. Glycerophospholipids were quantified in the cell pellet according to the procedure described previously by Klingler et al. [23]. Briefly, we added 1.3 mL methanol and 25 μg phosphatidylcholine dipentadecanoyl as internal standard to the cheek cell pellet, the suspension was placed in an ultrasound water bath (120 W, 35 kHz) for 20 min, and precipitated proteins were separated by centrifugation (3030× *g*, 20 min, 4 °C). Glycerophospholipids were transesterified into fatty acid methyl esters by adding 50 µL of sodium methoxide solution (Sigma-Aldrich, St. Louis, MO, USA) during 4 min at room temperature. Fatty acid methyl esters were quantified by gas chromatography using an SP-2560 capillary column (100 m × 0.25 mm i.d. × 20 μm) (Supelco, Sigma-Aldrich, St. Louis, MO, USA) in a Hewlett-Packard 6890 gas chromatograph (Agilent Technologies, Santa Clara, CA, USA) equipped with a flame ionization detector [29]. The temperature of the detector and the injector was 240 °C. The oven temperature was programmed at 175 °C for 30 min and increased at 2 °C/min to 230 °C and held at this temperature for 17 min. Helium was used as the inert carrier gas at a pressure of 45 psi. We used ChemStation software (version A.10.01., Agilent Technologies, Santa Clara, CA, USA) to analyse fatty acid data, and peaks were identified by comparing their retention times with appropriate fatty acid methyl ester standards (Sigma-Aldrich, St. Louis, MO, USA). The results are presented as weight percentages of all quantified fatty acids.

### 2.4. Design of the FFQ Validation

**Figure 1:**
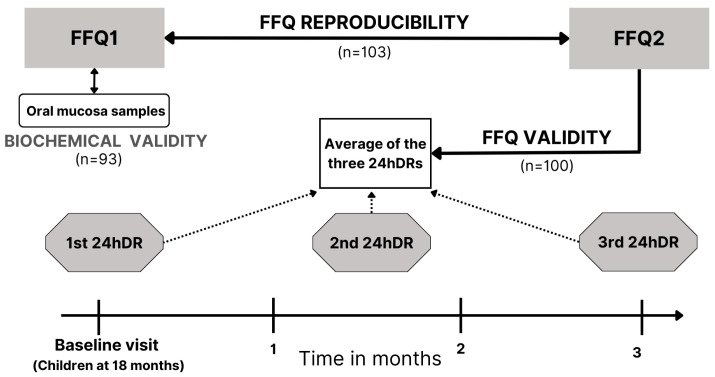
The design of the validation study among toddlers aged 18 months of the NELA study. FFQ, food frequency questionnaire; 24hDR, 24 h dietary recall.

Figure 1 shows information for the study design of the FFQ validation conducted during a 3-month period. The baseline interview took place when children were 18 months old, and parents provided information for the first FFQ, the first 24hDR, and the consent for oral mucosa sample collection. Approximately, in the middle of the period, we collected information for the second 24hDR, and by the end of the 3-month period, we collected information for the second FFQ and the third 24hDR.

To evaluate the reproducibility of the FFQ, we compared the food and nutrient intakes estimated by the same FFQ, after a 3-month period, under the assumption that children were under same comparability conditions. This period was considered adequate to avoid the effect of recent memory when answering the second FFQ and to capture usual dietary intakes without allowing the effect of rapid dietary changes that may occur in 18-month-old children.

The validity of the FFQ was assessed by comparing the usual nutrient intakes estimated by the second FFQ referred to the past three months with the average of nutrient intakes estimated by the three 24hDRs. We considered the three 24hDRs as the gold standard (reference method), as they were collected during the same period as the second FFQ was referring to. In addition, we compared fatty acid intake and fish consumption estimated by the first FFQ with the fatty acid profile determined from oral mucosa cells collected in the same baseline interview. We consider this second approach a complementary and novelty reference method to explore the relative biochemical validity of the FFQ.

### 2.5. Statistical Analysis

The main characteristics of the 18-month-old toddlers and their parents were described according to their participation in the validation study using mean and standard deviation (SD) for continuous variables and percentages for categorical variables. We used ANOVA and chi-square tests to compare characteristics of non-participants and participants on the validation study. We also calculated mean and standard deviation (SD) to describe the nutrient intakes and food group consumption, and we used paired Student’s *t*-test to compare them.

Prior to analyses, we conducted the Shapiro–Wilk test to assess the normality of energy and nutrient data, and the results indicated that the data were not normally distributed. Consequently, nutrient crude intakes from both FFQs and the three 24hDRs were log-transformed. In addition, energy-adjusted nutrient intakes were computed using the residual method proposed by Willett [4].

To explore FFQ reproducibility, we estimated Pearson correlation coefficients to compare nutrient and food group intakes reported from both FFQs. We also estimated the percentage of agreement of toddlers classified in the same or an adjacent quintile of nutrient crude intakes, according to cross-classification into quintiles of nutrient intakes estimated by the two FFQs.

To assess FFQ validity, we estimated Pearson correlation coefficients to compare nutrient intakes reported by the second FFQ and the average of the three 24hDRs, using log-transformed and energy-adjusted nutrient intakes. Because day-to-day variability (within variability) tends to attenuate the correlation between the FFQ and the 24hDR, de-attenuated Pearson correlation coefficients were calculated. The formula to calculate the de-attenuated correlations was the following:

(1) $$r\;de-attenuated=\sqrt{1+\{(\frac{S2w}{S2b})/n\}}$$

where *S*2*w* represents within-person variance and *S*2*b* between-person variance for each nutrient, and n is the number of replicated 24hDRs, in our case, n = 3 (30).

Additionally, we calculated Pearson correlation coefficients to explore the relative validity of the FFQ by comparing the fish consumption and fatty acid intakes estimated by the first FFQ with the percentage concentration of several fatty acids obtained from oral mucosa cells as conducted in previous validation studies comparing biomarkers with food and nutrient intakes [4,20].

All the statistical analyses were performed using STATA (version 16.1, StataCorp, College Station, TX, USA), and *p*-values < 0.05 were considered statistically significant.

## 3. Results

Table 1 shows the main characteristics of toddlers in the NELA study, differentiating between non-participants (n = 429) and participants (n = 103) in the validation study. Those toddlers who participated in the validation study were slightly younger (18.8 vs. 19.1 months, *p* = 0.02). In addition, participants’ fathers were significantly more likely to have university-level education (52.4% vs. 39.4%, *p* = 0.01).

### 3.1. Reproducibility

Table 2 shows the mean of daily food group consumption and Pearson correlation coefficients from the two FFQs. Overall, the consumption reported in both FFQs was similar for all food groups, although in the second FFQ, it was slightly higher for some food groups (e.g., eggs, fats, sweetened beverages, and water) and significantly lower for cereals and red meats. The average of correlation coefficients for log-transformed nutrients was r = 0.45, ranging from r = 0.20 for breads to r = 0.87 for legumes. The average of coefficient correlations for energy-adjusted food groups was r = 0.35, ranging from r = 0.13 for breads and fats to r = 0.58 for solid fats.

Table 3 shows mean daily nutrient intakes and Pearson correlation coefficients of the FFQ which was administered twice, 3 months apart. Means (SD) of energy and nutrient intakes estimated from both FFQs were similar, although the intake of some nutrients was slightly higher in the second FFQ (e.g., trans fatty acids, cholesterol, α-carotene, lycopene, and sodium). Significant correlation coefficients were observed for all log-transformed nutrients. The average of correlation coefficients for log-transformed nutrients was r = 0.49, ranging from r = 0.30 for sodium to r = 0.66 for iodine. Correlation coefficients varied for some nutrients after adjustment for energy, but all of them remained statistically significant. The average of coefficient correlations for energy-adjusted nutrients was r = 0.48, ranging from r = 0.28 for sodium intake to r = 0.62 for magnesium and zinc intakes. The average percentage of toddlers classified in the same or an adjacent quintile for nutrient crude intakes was 70.8%, ranging from 62.1% for β-cryptoxanthin to 81.6% for iodine.

### 3.2. Validity

Table 4 shows mean daily nutrient intakes and Pearson correlation coefficients for the second FFQ and the average of the three 24hDRs. Means (SD) of energy and nutrient intakes from the second FFQ were significantly higher than those estimated by the three 24hDRs for most of the nutrients (*p* < 0.05). The average of correlation coefficients for log-transformed nutrients was r = 0.45, ranging from r = 0.20 for α-carotene to r = 0.66 for retinol and vitamin D. When nutrient intakes were adjusted for energy, the average of the correlation coefficients was r = 0.48, ranging from r = 0.16 for α-carotene to r = 0.75 for vitamin E and improving also for macronutrients. The average of de-attenuated correlation coefficients was r = 0.52, ranging from r = 0.10 for sodium to r = 0.82 for vitamin E. The average percentage of toddlers classified in the same or an adjacent quintile in nutrient crude intakes was 70.6%, ranging from 54.0% for vitamin B6 to 84.0% for retinol.

Regarding the relative biochemical validity of the FFQ, Table 5 shows the coefficient correlations between the percentage of daily fatty acid intake and fish consumption from the first FFQ and the percentage of fatty acid concentrations in oral mucosa cells. We found statistically significant correlations between the percentage of docosahexaenoic acid (DHA, 22:6 n-3) estimated from the first FFQ with the same nutrient estimated in oral mucosa samples (r = 0.24, *p* = 0.021). We also found statistically significant correlations between the energy-adjusted consumption of total fish and the concentration of total omega-3 fatty acids (r = 0.31, *p* = 0.002) and DHA (r = 0.31, *p* = 0.002) as well as for consumption of white fish with total omega-3 fatty acids (r = 0.34, *p* = 0.0008) and DHA (r = 0.30, *p* = 0.004). No significant correlation coefficients were found between fish consumption and other fatty acids.

## 4. Discussion

This validation study shows a moderate reproducibility and validity of a short FFQ to assess usual diet during the previous 3 months of 18-month-old children in Spain, using three 24hDRs and the concentration of fatty acids in oral mucosa cells as a reference method. The reproducibility (mean r = 0.48) and validity (mean r = 0.48) of the FFQ were aligned with established thresholds for dietary assessment tools (r > 0.3).

To assess reproducibility, we compared nutrient intakes estimated from the same FFQ administered twice, three months apart. The average of correlation coefficients for FFQ reproducibility was r = 0.48 for energy-adjusted nutrient intakes and r = 0.35 for the intake of energy-adjusted food groups. These correlation coefficients were similar to those observed in previous studies by our group in pregnant women [30] and children aged 4 [20] and 7 years old [31]. However, comparison with other similar studies is limited due to the scarce number of FFQs validated in toddlers. A previous study with 296 participants aged 0–24 months old reported a higher average of correlation coefficients (r = 0.56) for the reproducibility of a 52-item FFQ. However, the FFQ was completed twice, one week apart, which is a very short period of time [13]. Another study carried out in 102 toddlers aged 12 to 35 months using an 89-item FFQ showed correlation coefficients ranging from r = 0.72 to r = 0.89 of reproducibility after two FFQs, four weeks apart [17]. It should be noted that the fact that both studies used a very short time interval between FFQ administration may have resulted in artificially higher correlation coefficients [4].

To assess validity, we used the average of three 24hDRs as a reference method, because the use of other assessments such as dietary records is burdensome and can generate losses to follow-up, among other problems (e.g., changes in usual diet). In our study, the correlation coefficients between the second FFQ and the average of the 24hDRs were moderately good, with an average of r = 0.48 for energy-adjusted nutrient intakes and most correlations within the range of the optimal cut-offs, as suggested by Willett (0.4 < r <0.7) [4] and Cade et al. (r > 0.3 or 0.4) [5]. These results are similar to those observed in other validation studies that also used 24hDR as a reference method to validate FFQs in children under 2 years [10,13,14,16,17]. These studies showed average correlation coefficients ranging from r = 0.29 in a study with 231 toddlers using a 68-item FFQ [16] to r = 0.53 in another study with 188 toddlers and a 94-item FFQ [14].

We also conducted a biochemical validation using the fatty acid content from oral mucosa cells as biomarkers of fatty acid intake. Plasma and erythrocyte lipids are used as biological markers for dietary fat intake [32]. High correlations have been reported for oral mucosa cells with these biological markers related to DHA and EPA content. In fact, strong correlation coefficients of r > 0·75 have been determined between DHA in oral mucosa cells, plasma, and erythrocyte glycerophospholipids [24]. Considering a lag phase of about 5 days, oral mucosa cells reflect short-term changes in dietary fat uptake, and they can be used alternatively to plasma and erythrocyte phospholipids as non-invasive n-3 fatty acid status markers [24]. In this study, we found a significant correlation between fish consumption and omega-3 fatty acid in oral mucosa, particularly for DHA, for which we also found a significant correlation for its intake. The observed weak correlation for blue fish consumption may reflect the low average intake (4.3 g/day). As far as we know, this is the first validation study of an FFQ using fatty acid measure in oral mucosa cells as a biomarker of dietary intake. However, our results are consistent with those observed in two other small sample studies with infants aged 12 months receiving formulas enriched with DHA that found good correlations between fatty acid profile from oral mucosa cells, plasma levels, and dietary intake of DHA [29,33].

Our findings suggest that a short FFQ may be a practical and feasible tool to be used in large-scale epidemiological studies with toddlers to assess usual diet, including nutrient, energy, and food intakes. Particularly, their use should be considered when resources and available time to collect the information is limited.

This study has both limitations and strengths. Among the limitations, the accuracy of dietary assessments may be influenced by the parents’ memory and their ability to estimate the portions consumed by their children. The reliance on parental recall may introduce bias, and future studies should explore objective methods such as direct observation to validate dietary intake. Nevertheless, the interviewer minimized the loss of memory by following the multiple-pass 24 h recall method [34]. Additionally, the use of 24hDR as a reference method could be questioned; however, when administered by expert interviewers using the multiple-pass 24 h recall method, it has been shown to provide similar estimations of energy and nutrients compared to other methods considered more accurate such as weighed food records or the doubly labelled water method [19,26]. We should also consider as study limitation the potential influence of changes in diet at 18 months of age and seasonality due to the use of a short 3-month period. Although these potential sources of variability could reduce FFQ reproducibility, we found satisfactory coefficient correlations for most nutrient and food intakes. Another potential limitation of this study may be the different sample size used for reproducibility and validity analyses, because we consider it more appropriate to maximize sample size and statistical power for every analysis. We repeated the analyses using only the 93 participants with complete information, and correlations for reproducibility and validity were very similar, although correlation coefficients were less robust but still significant for most nutrients. In addition, while the FFQ demonstrated moderate overall validity, lower correlations were observed for certain nutrients, such as sodium and vitamin B6. Lower correlations are commonly observed for some nutrients in validation studies, particularly when short FFQs are used. These low correlations may relate to different factors like the low sample size, the underreporting of some relevant foods when grouping food items, or the use of a low number of 24hDRs as the reference method that may not fully capture the intake for certain nutrients from foods less consumed. This lower performance should be further investigated to improve the overall FFQ validity. The use of glycerophospholipid from oral mucosal cells as a biomarker is a new approach for FFQ validation. However, the use of this reference method may have some limitations. Fatty acid composition can be influenced by individual metabolism and cell absorption differences [24]. In addition, while standardized protocols are used to ensure accuracy, collecting samples in toddlers can be challenging, such as requiring a cleaning of the toddler’s mouth before collection.

Regarding the strengths, participants in this validation study belong to a population-based cohort study, which allows the extrapolation of the results obtained to other Spanish toddlers. In addition, all dietary assessments were conducted by the same interviewer (MTPF), and all coding, as well as estimations of food weights and volumes from the 24hDR, were performed by the same experienced nutritionist (COB), which increases the accuracy and consistency of the nutritional data. Moreover, the analysis of fatty acid profile in oral mucosa cells as non-invasive biomarkers is a strength, as it offers an accurate and less invasive alternative to conventional methods that require blood samples [23,24]. This study’s use of oral mucosa biomarkers aligns with emerging non-invasive techniques, offering a scalable and child-friendly approach for dietary research.

## 5. Conclusions

This study shows that our short FFQ has moderate reproducibility and validity to assess usual intake of most nutrients and foods during the previous 3 months in 18-month-old Spanish children, using three 24hDRs and the fatty acid content in oral mucosa cells as a reference method. These results are consistent with other studies performed in toddlers and support that short FFQs may be a reliable and efficient method for assessing dietary intake in children under 3 years old.

## Figures and Tables

**Table 1:** Characteristics of toddlers and parents, stratified by participation (no/yes) in the validation study.

	Non-Participants (n = 429)	Participants (n = 103)	*p*-Value ^1^
Sex, n (%)			0.44
Male	211 (49.2)	55 (53.4)	
Female	218 (50.8)	48 (46.6)	
Age (months), mean (SD)	19.1 (1.1)	18.8 (0.8)	0.02
Maternal age (years), n (%)			0.74
≤29	85 (19.8)	17 (16.5)	
30–35	231 (53.9)	57 (55.3)	
≥36	113 (26.3)	29 (28.2)	
Maternal educational level, n (%)			0.92
Primary or less	71 (16.6)	16 (15.5)	
Secondary	105 (24.5)	24 (23.3)	
University	253 (59.0)	63 (61.2)	
Maternal social class, n (%)			0.83
High (I + II)	166 (38.7)	42 (40.8)	
Medium (III)	99 (23.1)	24 (23.3)	
Low (IV–V)	76 (17.7)	20 (19.4)	
Unemployed	88 (20.5)	17 (16.5)	
Paternal age (years), n (%)			0.84
≤29	53 (12.4)	12 (11.7)	
30–35	182 (42.4)	47 (45.6)	
≥36	194 (45.2)	44 (42.7)	
Paternal educational level, n (%)			0.01
Primary or less	122 (28.4)	16 (15.5)	
Secondary	138 (32.2)	33 (32.0)	
University	169 (39.4)	54 (52.4)	
Paternal social class, n (%)			0.55
High (I + II)	152 (35.4)	44 (42.7)	
Medium (III)	63 (14.7)	15 (14.6)	
Low (IV–V)	188 (43.8)	39 (37.9)	
Unemployed	26 (6.1)	5 (4.9)	

^1^ *p*-values were calculated by ANOVA test for continuous variables and χ^2^ test for categorical variables. SD, standard deviation; n, number.

**Table 2:** Mean daily food group intake and Pearson correlation coefficients of two FFQs in children aged 18 months old of the NELA study (n = 103).

Food Groups (Grams/Day)	FFQ1 ^1^	FFQ2 ^1^		Pearson Coefficient (FFQ1 vs. FFQ2)	
	Mean (SD)	Mean (SD)	*p*-Value ^2^	R ^3^	r adj ^4^
Dairy products	632.1 (277.7)	603.9 (256.2)	0.36	0.42	0.45
Eggs	15.2 (8.2)	17.8 (6.8)	<0.001	0.54	0.56
Meat and processed meats	35.3 (10.5)	35.4 (15.6)	0.97	0.29	0.16
White meat	18.6 (6.1)	17.8 (4.3)	0.23	0.25	0.18
Red meat	10.6 (6.3)	9.0 (6.1)	0.02	0.30	0.24
Processed meat	6.1 (5.4)	8.6 (13.0)	0.06	0.53	0.22
Fish	18.8 (9.1)	20.1 (9.2)	0.17	0.54	0.48
White fish	14.5 (7.2)	14.8 (6.1)	0.66	0.55	0.52
Blue fish	4.3 (5.3)	5.3 (6.6)	0.11	0.58	0.42
Vegetables	127.9 (66.1)	120.3 (59.2)	0.26	0.53	0.38
Pulse	16.4 (6.8)	15.0 (6.3)	0.07	0.87	0.30
Fruit	207.4 (121.5)	204.5 (139.1)	0.84	0.35	0.44
Breads	32.0 (29.2)	37.0 (25.8)	0.17	0.20	0.13
Cereals	28.2 (11.9)	24.9 (11.2)	0.01	0.42	0.52
Potatoes	38.1 (20.6)	34.6 (20.0)	0.16	0.29	0.21
Pre-prepared food	12.0 (10.9)	14.0 (12.4)	0.12	0.37	0.40
Fats	11.2 (6.4)	13.1 (6.4)	0.02	0.22	0.13
Oils	11.0 (6.2)	12.8 (6.5)	0.03	0.22	0.15
Solid fats	0.1 (0.7)	0.3 (0.9)	0.04	0.60	0.58
Sweets and sugar	12.7 (10.5)	14.6 (12.4)	0.13	0.60	0.36
Sweetened beverages	9.7 (34.3)	16.3 (31.0)	0.01	0.57	0.56
Water (as a drink)	326.3 (159.5)	386.3 (181.1)	<0.001	0.33	0.27
Average				0.44	0.35

^1^ FFQ1 and FFQ2, the same FFQ was administered at baseline (FFQ1) and 3 months later (FFQ2). ^2^ *p*-value from paired *t*-tests; ^3^ r, correlation coefficients after food group intakes were log-transformed; ^4^ r adj, correlation coefficients using food group intakes adjusted for total energy. FFQ, food frequency questionnaire.

**Table 3:** Mean daily nutrient intake and Pearson correlation coefficients of two FFQs in toddlers aged 18 months old of the NELA study (n = 103).

Nutrient Intake (Units/Day)	FFQ1 ^1^	FFQ2 ^1^		Pearson Coefficient (FFQ1 vs. FFQ2)		% of Agreement ^5^
	Mean (SD)	Mean (SD)	*p*-Value ^2^	R ^3^	r adj ^4^	
Energy (kcals)	1194.0 (239.4)	1205.5 (233.7)	0.63	0.46	-	63.1
Protein (g)	47.4 (8.9)	48.6 (9.6)	0.21	0.46	0.45	68.9
Total carbohydrates (g)	146.4 (35.5)	143.3 (34.3)	0.40	0.38	0.48	68.0
Dietary fiber (g)	12.3 (4.0)	11.9 (4.0)	0.28	0.40	0.49	67.0
Total fat (g)	49.2 (12.7)	51.1 (12.4)	0.09	0.56	0.49	70.9
SFA (g)	18.1 (5.3)	18.6 (5.0)	0.26	0.58	0.47	72.8
MUFA (g)	21.6 (6.2)	22.8 (6.3)	0.05	0.49	0.32	67.0
PUFA (g)	5.9 (1.7)	5.9 (1.9)	0.82	0.48	0.33	71.8
Omega-3 (g)	0.7 (0.2)	0.8 (0.3)	0.13	0.53	0.45	70.9
Omega-6 (g)	5.0 (1.5)	5.0 (1.7)	0.85	0.45	0.32	70.9
Trans fatty acid (g)	0.4 (0.2)	0.5 (0.2)	0.02	0.57	0.59	74.8
Cholesterol (mg)	167.1 (45.3)	176.1 (38.2)	0.03	0.52	0.35	75.7
Retinol (µg)	425.2 (168.7)	394.1 (167.0)	0.08	0.48	0.45	68.9
α-Carotene (µg)	169.3 (110.0)	202.0 (125.9)	0.00	0.59	0.58	74.8
β-Carotene (µg)	1530.2 (688.0)	1573.6 (792.7)	0.56	0.48	0.47	71.8
β-Cryptoxanthin (µg)	97.1 (68.9)	116.3 (88.6)	0.05	0.36	0.29	62.1
Lutein-Zeaxanthin (µg)	2137.4 (1111.4)	1831.3 (863.3)	0.01	0.49	0.40	74.8
Lycopene (µg)	764.2 (491.3)	1000.4 (639.7)	0.00	0.44	0.53	65.1
Vitamin B6 (mg)	1.2 (0.3)	1.1 (0.3)	0.06	0.48	0.59	75.7
Folate (µg/day)	184.7 (43.7)	179.1 (45.7)	0.22	0.46	0.44	70.9
Vitamin B12 (µg)	3.7 (1.1)	3.8 (1.1)	0.18	0.56	0.57	69.9
Vitamin C (mg)	74.4 (29.9)	72.4 (36.7)	0.58	0.35	0.29	64.1
Vitamin D (µg)	5.0 (2.0)	5.2 (2.2)	0.37	0.62	0.57	68.9
Vitamin E (mg)	7.3 (3.4)	6.7 (3.4)	0.06	0.52	0.58	73.8
Calcium (mg)	991.2 (290.1)	997.8 (295.2)	0.83	0.51	0.58	72.8
Iron (mg)	11.8 (5.6)	9.8 (5.1)	0.00	0.53	0.61	72.8
Magnesium (mg)	203.9 (43.8)	200.9 (46.8)	0.54	0.46	0.62	76.7
Potassium (mg)	2190.7 (487.4)	2179.0 (515.7)	0.82	0.48	0.55	75.7
Sodium (mg)	1180.2 (266.1)	1275.2 (319.3)	0.01	0.30	0.28	68.0
Zinc (mg)	6.8 (1.5)	6.5 (1.6)	0.11	0.47	0.62	70.9
Iodine (µg)	111.2 (53.2)	111.2 (53.3)	0.34	0.66	0.60	81.6
Total water (g)	1314.5 (28.8)	1361.3 (28.8)	0.14	0.43	0.38	65.1
Average				0.49	0.48	70.8

^1^ FFQ1 and FFQ2, the same FFQ was first administered at baseline (FFQ1) and the second (FFQ2) 3 months later. ^2^ *p*-value from paired *t*-tests. ^3^ r, correlation coefficients after log-transforming nutrient crude intakes. ^4^ r adj, correlation coefficients using nutrient intakes adjusted for total energy. ^5^ Percentage of children classified in the same or adjacent quintile in nutrient crude intakes. FFQ, food frequency questionnaire; MUFA, monounsaturated fatty acid; PUFA, polyunsaturated fatty acid; SFA, saturated fatty acid.

**Table 4:** Mean daily nutrient intake and Pearson correlation coefficients between FFQ2 and the average of three 24hDRs ^1^.

Nutrient Intake (Units/Day)	FFQ2 ^2^	24hDRav ^3^		Pearson Coefficient (FFQ2 vs. 24hDRav)			% of Agreement ^8^
	Mean (SD)	Mean (SD)	*p*-Value ^4^	R ^5^	r adj ^6^	r de-att ^7^	
Energy (kcals)	1209.5 (233.6)	1056.3 (125.6)	<0.001	0.45	-	-	69.0
Protein (g)	48.7 (9.6)	46.4 (9.5)	0.04	0.33	0.57	0.60	66.0
Total carbohydrates (g)	143.7 (34.6)	128.6 (19.6)	<0.001	0.42	0.50	0.54	71.0
Dietary fiber (g)	11.9 (4.1)	9.8 (3.0)	<0.001	0.38	0.53	0.59	75.0
Total fat (g)	51.4 (12.4)	41.5 (7.8)	<0.001	0.59	0.51	0.55	75.0
SFA (g)	18.7 (5.0)	15.8 (3.5)	<0.001	0.61	0.48	0.54	72.0
MUFA (g)	22.9 (6.3)	17.0 (3.5)	<0.001	0.48	0.33	0.36	69.0
PUFA (g)	5.9 (1.9)	5.2 (1.3)	<0.001	0.52	0.39	0.41	76.0
Omega-3 (g)	0.8 (0.3)	0.7 (0.2)	<0.001	0.48	0.43	0.47	76.0
Omega-6 (g)	5.0 (1.7)	4.4 (1.1)	<0.001	0.45	0.31	0.32	73.0
Trans fatty acid (g)	0.5 (0.2)	0.3 (0.2)	<0.001	0.48	0.66	0.71	77.0
Cholesterol (mg)	176.9 (37.7)	151.0 (43.0)	<0.001	0.37	0.28	0.30	64.0
Retinol (µg)	391.8 (167.0)	377.9 (143.1)	0.28	0.66	0.70	0.75	84.0
α-Carotene (µg)	205.8 (126.6)	786.5 (589.2)	<0.001	0.20	0.16	0.32	61.0
β-Carotene (µg)	1579.3 (805.1)	2010.4 (1253.4)	<0.001	0.35	0.30	0.31	69.0
β-Cryptoxanthin (µg)	116.4 (89.4)	60.6 (85.7)	<0.001	0.30	0.34	0.30	69.0
Lutein-Zeaxanthin (µg)	1810.5 (844.1)	1062.0 (1015.8)	<0.001	0.35	0.24	0.43	61.0
Lycopene (µg)	1014.5 (643.7)	1069.6 (978.8)	0.54	0.24	0.33	0.36	66.0
Vitamin B6 (mg)	1.1 (0.3)	1.1 (0.3)	0.87	0.21	0.37	0.43	54.0
Folate (µg/day)	179.2 (46.3)	136.5 (31.2)	<0.001	0.37	0.39	0.41	68.0
Vitamin B12 (µg)	3.8 (1.1)	3.7 (1.6)	0.24	0.52	0.43	0.39	75.0
Vitamin C (mg)	72.4 (37.4)	55.4 (24.7)	<0.001	0.45	0.33	0.37	65.0
Vitamin D (µg)	5.2 (2.2)	4.9 (2.3)	0.22	0.66	0.69	0.76	76.0
Vitamin E (mg)	6.6 (3.3)	5.5 (3.0)	<0.001	0.61	0.75	0.82	72.0
Calcium (mg)	996.2 (292.8)	884.4 (252.3)	<0.001	0.61	0.69	0.76	77.0
Iron (mg)	9.7 (5.0)	8.2 (3.5)	<0.001	0.61	0.63	0.72	81.0
Magnesium (mg)	201.0 (46.8)	171.5 (29.2)	<0.001	0.32	0.56	0.61	69.0
Potassium (mg)	2184.9 (522.1)	2008.1 (352.5)	0.001	0.32	0.56	0.62	65.0
Sodium (mg)	1281.4 (320.3)	1031.9 (269.9)	<0.001	0.25	0.29	0.10	59.0
Zinc (mg)	6.5 (1.5)	5.9 (1.2)	<0.001	0.52	0.69	0.75	74.0
Iodine (µg)	115.9 (52.3)	98.6 (44.9)	<0.001	0.58	0.67	0.74	75.0
Total water (g)	1358.3 (29.5)	1447.3 (28.6)	0.001	0.61	0.72	0.79	76.0
Average				0.45	0.48	0.52	70.6

^1^ Three participants were excluded from the analyses for incomplete data on 24hDRs. ^2^ FFQ2, second food frequency questionnaire. ^3^ 24hDRav, average of the three 24 h dietary recalls. ^4^ *p*-value from paired t-tests. ^5^ r, correlation coefficients after log-transforming nutrient crude intakes. ^6^ r adj, correlation coefficients using nutrient intakes adjusted for total energy. ^7^ r de-att, de-attenuated correlation coefficients using nutrient intakes adjusted for total energy. ^8^ Percentage of children classified in the same or an adjacent quintile in nutrient crude intakes. 24hDR, 24 h dietary recall; FFQ, food frequency questionnaire; MUFA, monounsaturated fatty acid; PUFA, polyunsaturated fatty acid; SFA, saturated fatty acid.

**Table 5:** Pearson correlation coefficients between percentage of daily fatty acid intake and fish consumption from the first FFQ and percentage of oral fatty acids in children aged 18 months old of the NELA study (n = 93) ^1^.

	Fatty Acid Intake (%) ^2^		Total Fish ^3^		White Fish ^3^		Blue Fish ^3^	
Fatty Acids	R ^4^	r adj ^5^	R ^4^	r adj ^5^	R ^4^	r adj ^5^	R ^4^	r adj ^5^
SFA	−0.07	−0.13	−0.04	−0.05	−0.12	−0.13	0.15	0.05
MUFA	−0.07	−0.12	0.10	0.10	0.14	0.14	−0.17	−0.00
PUFA	−0.19	−0.20	−0.01	0.03	0.05	0.07	−0.20	−0.05
*Trans*	0.05	0.06	0.14	0.13	0.19	0.15	0.12	0.00
Omega-3	0.12	0.10	0.31 *	0.31 *	0.34 *	0.34 *	0.09	0.09
Omega-6	−0.18	−0.20	−0.05	−0.01	0.01	0.03	−0.23	−0.06
EPA (20:5)	0.15	0.09	0.12	0.13	0.13	0.13	−0.07	0.10
DHA (22:6)	0.24 *	0.20	0.31 *	0.31 *	0.30 *	0.30 *	0.24	0.12

^1^ Fish consumption (from the first food frequency questionnaire) and oral mucosa cell samples were collected the same day. ^2^ Percentage of daily fatty acid intake from the first FFQ. ^3^ Fish consumption is expressed in grams/day. ^4^ r, coefficient correlations after log-transformed fish consumption and fatty acids from oral mucosa cells. ^5^ r adj, correlation coefficients using fish consumption adjusted for total energy intake and log-transformed oral fatty acids. * *p*-value < 0.05. DHA, docosahexaenoic acid; EPA, eicosapentaenoic acid; MUFA, monounsaturated fatty acid; PUFA, polyunsaturated fatty acid; SFA, saturated fatty acid.

## Data Availability

Any request of data, codebook, and analytic code of this article will be passed to members of the Nutrition in Early Life and Asthma Committee for deliberation. However, the data sets generated and analysed in the current study are not expected to be available outside the Nutrition in Early Life and Asthma due to the participants’ consent forms and ethics approvals did not included permission for open access.

## Supplementary Materials

The following supporting information can be downloaded at: https://www.mdpi.com/article/10.3390/nu16244403/s1, Table S1: Description of the food items integrated into the food groups. List of the names of other members of the NELA study group and EPINUT group.
