# Supplementary material for: Validity of a Short Food Frequency Questionnaire for Toddlers of NELA Birth Cohort Study
Source: Nutrients. 2024 Dec 22;16(24):4403. doi: 10.3390/nu16244403 (PMC11677162; doi:10.3390/nu16244403)
Supplement: Supplementary file 1 [file nutrients-16-04403-s001.zip › nutrients-3354538-supplementary.pdf]

Supplemental table S1 from the article entitled **Reproducibility and validity of a short food frequency questionnaire for dietary assessment in children aged 18 months: the NELA birth cohort study.**

Supplemental table S1. Description of the food items integrated into the food groups.

Dairy products	Breast milk; Follow-on formula (L2); Growing-up formula (L3); Whole cow's milk; Semi-skimmed cow's milk; Milkshakes; Yogurt; Cheese (all types); Dairy desserts (custard, flan, pudding, milk ice cream)
Eggs	Eggs
Meat and processed meats	Chicken or turkey; Beef, pork, lamb; Processed meat (ham, sausage, chorizo, etc.)
White Meat	Chicken or turkey
Red Meat	Beef, pork, lamb
Processed Meat	Processed meat (ham, sausage, chorizo, etc.)
Fish	White fish (hake, sole, sea bream, whiting or similar); Large blue fish (swordfish, tuna, bonito); Small blue fish (anchovy, sardine, salmon, mackerel)
White fish	White fish (hake, sole, sea bream, whiting or similar)
Blue fish	Large blue fish (swordfish, tuna, bonito or similar); Small blue fish (anchovy, sardine, salmon, mackerel or similar)
Vegetables	Raw vegetables (lettuce, tomato, carrot, cucumber, etc.); Cooked vegetables (zucchini, broccoli, cauliflower, etc.)
Legumes	Lentils, chickpeas, beans, peas
Fruit	Oranges; Banana, apple, or pear; Other fruits (peach, strawberry, kiwi, melon, etc.)
Breads	White bread; Wholemeal bread
Cereals	Cereals added to milk, yogurt, or porridge; Rice or pasta
Potatoes	Homemade potatoes (boiled, fried, baked, mashed); Frozen French fries
Pre-prepared food	Snacks (puff snacks, potato chips, and similar); Pizza, pastry, or similar
Fats	Extra virgin olive oil; Other oils such as sunflower; Butter or margarine
Oils	Extra virgin olive oil; Other oils such as sunflower;
Solid fats	Butter or margarine
Sweets and sugar	Cookies (all types); Pastries (such as muffins, croissants, cakes, sweets, etc.); Chocolates (all types); Candies, sweets, treats; Sugar, honey, jams
Sweetened beverages	Sugar-sweetened beverages; Artificial-sweetened beverages; Packaged fruit juices
Water	Water

[†]**Other members of the NELA Study Group:** L Garcia-Marcos, ME Candel-Torralba, MJ Gimenez-Banon, A Martinez-Torres, V Perez-Fernandez, M Sanchez-Solis, A Nieto, MT Prieto-Sanchez, M Sanchez-Ferrer, L Fernanez-Palacios, VP Gomez-Gomez, C Martinez-Gracia, P Peso-Echarri, G Ros-Berruezo, M Santaella-Pacual, A Gazquez, M Sanchez-Campillo, A Serrano-Munuera, M Zornoza-Moreno, P Jimenez-Guerrero, E Adomnei, JJ Arense-Gonzalo, J Mendiola, F Navarro-Lafuente, AM Torres-Cantero, M Segovia-Hernandez, G Yagüe-Guirao, PL Valero- Guillen, FV Aviles-Plaza, J Cabezas-Herrera, A Martinez- Lopez, M Martinez-Villanueva, JA Noguera-Velasco, A Franco-Garcia, AM Garcia- Serna, T Hernandez-Caselles, E Martin-Orozco, M Norte-Muñoz, M Canovas, E Cantero-Cano, T de Diego, JM Pastor, RA Sola-Martínez, A Esteban-Gil, JT Fernandez-Breis. MV Alcantara, S Hernandez, C Lopez-Soler. **Other members of the EPINUT Group:** M. García-de-la-Hera, L. Torres-Collado, A.J. Signes-Pastor, M.d.C Esquiva-Antolino.
